# Association between hemoglobin dynamic trajectories and 28-day mortality in elderly patients with sepsis: A retrospective cohort study using the MIMIC-IV database

**DOI:** 10.1371/journal.pone.0327443

**Published:** 2026-05-04

**Authors:** Yan Zeng, Jun Wang, Caoyi Liu, Jingwei Zhang

**Affiliations:** 1 Department of Hematology, West China School of Medicine, Sichuan University, Sichuan University affiliated Chengdu Second People’s Hospital, Chengdu Second People’s Hospital, Chengdu, China; 2 Department of BIood Transfusion, West China School of Medicine, Sichuan University, Sichuan University affiliated Chengdu Second People’s Hospital, Chengdu Second People’s Hospital, Chengdu, China; Pescara General Hospital, ITALY

## Abstract

**Background:**

Anemia is a common complication in sepsis and is associated with poor prognosis. However, there are few studies on the dynamic changes in hemoglobin (Hb) levels and their relationship with prognosis in elderly patients with sepsis. This study aims to retrospectively investigate the relationship between Hb trajectories and 28-day mortality in this population.

**Methods:**

This retrospective observational study used data from 4,961 elderly patients with sepsis extracted from the MIMIC-IV database(2008–2019). Hb trajectories were identified using a Joint Latent Class Model (JLCM) for longitudinal pattern classification. Following validation of proportional hazards assumptions (Schoenfeld residual test), we assessed the prognostic relationship between Hb trajectories and 28-day mortality using multivariable Cox regression models and Kaplan-Meier survival curves. Sensitivity analyses included sex-stratified multivariable Cox regression, subgroup analysis, and E-value assessment.

**Results:**

Hb trajectories were classified into three patterns: Class 1 with persistently low levels, Class 2 with medium levels and a gradual decline, and Class 3 with a rapid decline from higher levels. Using Class 2 as the reference, multivariable Cox regression showed that Class 1 (HR = 1.38, 95% CI = 1.18–1.62, p < 0.001) and Class 3 (HR = 1.21, 95% CI = 1.03–1.42, p = 0.020) were associated with higher 28-day mortality. Kaplan-Meier curves indicated the lowest survival probability in Class 1 and the highest in Class 2. Gender-specific analysis revealed consistent trajectory patterns between males and females, but higher Hb values in males. The predictive power of the rapid decline pattern for early mortality was stronger in males.

**Conclusion:**

The Hb trajectories in elderly sepsis patients are significantly associated with early mortality. Persistently low levels and rapid declines in Hb are indicators of poor prognosis.

## Introduction

Sepsis, a systemic inflammatory response syndrome induced by infection, represents one of the most prevalent and lethal conditions encountered in the Intensive Care Unit (ICU), marked by significant morbidity and mortality rate [1]. Although advancements in the early detection and therapeutic approaches for sepsis have been made in recent years, the mortality rate among sepsis patients remains alarmingly high. Key objectives in the management of sepsis within the ICU include the early evaluation of patient prognosis, prompt identification of high-risk individuals, and the implementation of effective interventions [2]. Anemia is a frequently observed complication in patients with sepsis, with an incidence rate of 58% during ICU hospitalization. Among critically ill patients whose ICU stay extends beyond seven days, 71.8% experience varying degrees [3,4]

Hemoglobin (Hb) is the most frequently utilized biomarker for evaluating the presence and severity of anemia. It not only serves as an indicator of a patient’s hematological status but is also associated with the severity of sepsis, multiple organ failure, and adverse prognostic outcomes [5,6]. Nonetheless, prior research has predominantly concentrated on the baseline values of static Hb levels, thereby neglecting the dynamic variations of Hb during sepsis treatment. These dynamic changes in Hb, akin to platelet parameters, may provide a more precise reflection of the clinical progression and mortality risk in sepsis patients, as evidenced by their acute decline, short-term fluctuations, or long-term stability [7–9]. Current studies have indicated that in populations without traumatic brain injuries, dynamic changes in Hb levels correlate with mortality in patients who do not have sepsis [10]. Dynamic Hb trajectories and prognosis varies among populations with different underlying diseases and complications. A recent study by Huang et al. identified three Hb trajectories in sepsis patients and associated rapid decline with adverse outcomes, providing foundational insights into Hb dynamics [11]. However, this correlation is not focused on an elderly population with sepsis, which exhibits distinct immune dysregulation and frailty. The incidence and mortality rates of sepsis significantly increase with age in the elderly [12]. Elderly individuals at high risk, due to factors such as frailty, complex underlying diseases, long-term chronic anemia, or malnutrition, may exhibit distinct hematological characteristics compared to the general population [13]. Nevertheless, there remains a lack of systematic research examining the correlation between Hb change trajectories and early mortality in elderly patients with sepsis. Therefore, exploring the relationship between the dynamic trajectory of Hb changes and the prognosis of elderly patients with sepsis is not only clinically significant but also contributes to enhancing the prognostic evaluation system for sepsis.

This study aims to explore the association between dynamic changes in Hb levels among elderly sepsis patients, based on data from the Medical Information Mart for Intensive Care (MIMIC-IV, version 3.0), and 28-day mortality. The findings provide new insights for early risk assessment in this demographic through a retrospective analysis.

## Materials and methods

### Population and data collection

This study utilized data from MIMIC-IV, version 3.0, which includes information on over 70,000 patients admitted to the ICUs of Beth Israel Deaconess Medical Center in Boston, MA, from 2008 to 2019 [14]. Access to the database required completion of the “CITI Data or Specimens Only Research” training course available on the National Institutes of Health website, and approval was granted to extract data for research purposes (Certificate No for Zhang: ID: 65677952). The inclusion criteria were as follows: (1) patients met the diagnostic criteria for sepsis according to the Sepsis-3 guidelines, specifically requiring a suspected infection and a change of at least 2 points in the Sequential Organ Failure Assessment (SOFA) score [9]; (2) patients had baseline platelet count data at the time of admission and at least three platelet count measurements recorded within 28 days of admission; (3) age ≥ 65 years. Patients were excluded from the study if they met any of the following criteria: (1) repeated ICU admissions; (2) an admission duration of less than 72 hours. Patients with multiple ICU admissions during the same hospitalization were excluded to prevent violation of statistical independence assumptions, eliminate confounding from inter-admission interventions, and align with standard analytic approaches for trajectory modeling [8,15].

Structured Query Language (SQL) with PostgreSQL (version 13.0) and Navicat software (version 17.0) were employed to identify the cohort and extract pertinent clinical data [16]. Demographic information, comorbidities, and laboratory indicators were sourced from the MIMIC-IV database. The Hb laboratory data encompassed measurements taken on days 1, 3, 5, and 7 post-admission to the ICU, as well as the averages for weeks 2, 3, and 4, including the minimum, maximum, and average values recorded during hospitalization. In instances where data for days 3, 5, or 7 were absent, values from the nearest available day were utilized to bridge the gaps. Hb values were anchored to seven pre-specified study days with days 1,3,5,7, using the first measurement within ±24-hour window and days 14,21,28, using the average measurement for weeks 2, 3, and 4 post admission. Missing values were retained as such in trajectory modeling without imputation to avoid introducing artificial trend [17]. Missing data were detailed in S1 Table, which is included in the section on Clinical Characteristics of Different Hb Trajectories. Other laboratory tests extracted maximum, minimum, or average values during hospitalization based on their clinical relevance. The primary outcome of the study was 28-day mortality. Variables with missing data exceeding 10% were excluded from the analysis, in accordance with the recommendations of the Strengthening the Reporting of Observational Studies in Epidemiology (STROBE) statement [18]. Multiple imputation was employed to address variables with missing data of less than 10% [19]. The patients ultimately included will be classified according to their Hb trajectory patterns, and a follow-up analysis of their 28-day mortality will be performed.

### Identification of different trajectories

We utilized the R package lcmm (version 2.0.2) to identify subpopulations exhibiting heterogeneous Hb trajectories [8]. A joint latent class model (JLCM)was employed for the longitudinal analysis. The JLCM integrates repeatedly measured biomarkers and outcome variables across different time dimensions. The JLCM posits that the population comprises multiple latent classes, each characterized by a shared mean trajectory [17].. By explicitly capturing population heterogeneity, it avoids the information loss inherent in two-stage modeling and has become a widely used tool for analyzing dynamic data characteristics [20]. Additionally, we implemented a parametric survival model, where the baseline hazard function adhered to a class-specific Weibull distribution [8]. The optimal number of classes was established by minimizing the log-likelihood, alongside evaluating the Akaike Information Criterion (AIC), Bayesian Information Criterion (BIC), sample-adjusted BIC (SABIC), entropy closest to 1, balanced class proportions, and average posterior probability [10,21].

### Sample size consideration

As a retrospective cohort study utilizing real-world data from the MIMIC-IV database, our analytical sample size was determined by all eligible patients meeting predefined criteria. Formal a priori power calculation is typically infeasible in such designs due to unknown event rates and covariate distributions before data extraction. We implemented two complementary approaches to validate statistical adequacy. Firstly, the final cohort included 4,961 patients with 987 mortality events (19.89%). With 11 covariates in the fully adjusted Cox model, the EPV ratio was 89.7 (≈90), substantially exceeding the recommended threshold of EPV ≥ 10 [17]. This ensures reliable multivariable regression calibration. Secondly, the sample size adequacy was verified using the “pmsampsize” R package (v1.0.1). Key parameters included an anticipated model discrimination (C-statistic) of 0.75, an outcome prevalence of 19.89%, 12 candidate predictor variables and the margin of error for the estimation of the model intercept (≤0.05). The calculation yielded a minimum requirement of 803 participant. The events per variable (EPV) ratio was 90, exceeding the minimum requirement of EPV [22].

### Ethical approval and consent to participate

This study was performed in compliance with local and institutional ethical regulations. As a retrospective analysis using anonymized public database data, ethical approval was not required, and written informed consent was waived in accordance with national laws and institutional policies. All data utilized in this research were obtained from the publicly accessible, de-identified MIMIC-IV database, ensuring the privacy and confidentiality of all included participants were fully protected.

### Statistical analysis

Continuous variables were represented by means for normally distributed data and by medians for skewed data, with group comparisons conducted using the Kruskal-Wallis test. Categorical variables were expressed as percentages (%), with group comparisons performed using the chi-squared test or Fisher’s exact test. Univariate and multivariate Cox regression analyses were utilized to assess the association between characteristic variables and 28-day mortality. The criteria for adjusting covariates in the multivariate analysis were informed by prior literature, clinical experience, and the outcomes of univariate Cox regression [23]. The covariate selection principles were as follows: (1) After adjustment, if the original hazard ratio (HR) effect of the independent variable on the outcome variable changes by more than 5%; (2) There is no collinearity between the covariates and the independent variable or among the covariates themselves. Collinearity is assessed using the Variance Inflation Factor (VIF), with a VIF of 10 or greater indicating the presence of collinearity. Four models were established using multivariate Cox regression to analyze the association between trajectories and 28-day mortality. The Schoenfeld residual test was employed to verify the assumption of proportional hazards in the Cox analysis [24]. Survival curves were plotted using Kaplan-Meier (KM) analysis, and log-rank tests were conducted. Subgroup analysis and variable regrouping were employed as part of the sensitivity analysis. The E-value for sensitivity analyses and subgroup analysis was utilized to assess the robustness of the observed associations [25]. All analyses were conducted using R Statistical Software (Version 4.2.2, available at http://www.R-project.org, The R Foundation) and the Free Statistics analysis platform (Version 2.1.1, Beijing, China). A two-tailed test was performed, with a significance level set at p < 0.05 [26].

## Results

### Baseline characteristics

This study included a total of 4,961 participants, who were divided into two groups based on whether death occurred within 28 days: the survival group (n = 3,974) and the death group (n = 987). The process of participant inclusion, following the established inclusion and exclusion criteria, is illustrated in S1 Fig. A comparison of the basic characteristics and clinical indicators among the study subjects is presented in Table 1. Compared to the survival group, patients in the death group were significantly older (78.3 ± 7.9 years vs. 76.2 ± 7.4 years, p < 0.001) and exhibited significantly higher severity of illness, as indicated by APACHE III, SAPS II, and SOFA scores. Multiple blood laboratory indicators (such as white blood cell count, platelet count, urea nitrogen, creatinine, and lactate) as well as physiological indicators (including heart rate, blood pressure, oxygen saturation, and blood glucose) demonstrated significant differences between the two groups, indicating a poorer health status in the deceased group. Furthermore, the prevalence of heart failure, cerebrovascular disease, and liver and kidney diseases was higher in the deceased group (all p < 0.01), and the Charlson Comorbidity Index was also elevated in this group, revealing a greater burden and complexity of diseases.

**Table 1:** Baseline characteristics of the study population.

Variables	Total (n = 4961)	Survival group (n = 3974)	Death group (n = 987)	p
Age	76.6 ± 7.5	76.2 ± 7.4	78.3 ± 7.9	< 0.001
Gender, n (%)				0.314
Female	2910 (58.7)	2345 (59)	565 (57.2)	
Male	2051 (41.3)	1629 (41)	422 (42.8)	
Hb1	10.9 ± 1.9	10.9 ± 1.8	10.7 ± 2.1	0.004
Heart rate-mean (bpm)	84.8 ± 15.1	84.2 ± 14.4	87.2 ± 17.5	< 0.001
SBP-mean (mmHg)	113.8 ± 13.0	113.9 ± 12.7	113.0 ± 14.2	0.044
DBP-mean (mmHg)	57.8 ± 9.0	57.6 ± 8.8	58.5 ± 9.6	0.006
Resp rate-mean (bpm)	19.7 ± 3.8	19.3 ± 3.7	21.1 ± 4.3	< 0.001
Temperature-mean (°C)	36.8 ± 0.6	36.8 ± 0.5	36.8 ± 0.7	0.003
SpO2-mean (%)	97.2 ± 2.0	97.3 ± 1.8	96.8 ± 2.3	< 0.001
Glucose-mean (mg/dL)	137.0 (120.6, 166.4)	135.5 (120.8, 162.4)	146.0 (120.0, 186.8)	< 0.001
Platelets-min (×10^9^/L)	146.0 (102.0, 206.8)	143.0 (101.0, 202.0)	159.0 (104.0, 226.0)	< 0.001
WBC-max (×10^9^/L)	15.0 (11.0, 20.2)	14.9 (11.0, 20.1)	15.7 (10.9, 20.6)	0.149
Anion gap-max (mmol/L)	16.0 (13.0, 19.0)	16.0 (13.0, 19.0)	18.0 (15.0, 21.0)	< 0.001
Bicarbonate-min (mmol/L)	20.3 ± 5.0	20.5 ± 4.9	19.6 ± 5.6	< 0.001
BUN-max (mg/dL)	27.0 (19.0, 45.0)	25.0 (18.0, 41.0)	38.0 (25.0, 58.0)	< 0.001
Calcium-min (mg/dL)	7.9 ± 0.9	7.9 ± 0.9	7.9 ± 0.9	0.226
Chloride-min (mmol/L)	102.8 ± 6.6	103.1 ± 6.4	101.7 ± 7.2	< 0.001
Creatinine-max (mg/dL)	1.3 (0.9, 2.2)	1.3 (0.9, 2.0)	1.7 (1.1, 2.6)	< 0.001
Sodium-min (mmol/L)	137.0 ± 5.2	137.0 ± 5.0	137.2 ± 6.0	0.328
Potassium-max (mmol/L)	4.8 ± 0.8	4.7 ± 0.8	4.9 ± 0.9	< 0.001
INR-max (s)	1.4 (1.2, 1.7)	1.4 (1.2, 1.7)	1.5 (1.2, 2.0)	< 0.001
PT-max (s)	15.6 (13.6, 18.6)	15.5 (13.6, 18.2)	16.2 (13.7, 21.8)	< 0.001
APTT-max (s)	35.3 (29.7, 49.8)	34.9 (29.6, 47.7)	37.9 (30.3, 58.1)	< 0.001
Lactate-max (mmol/L)	2.4 (1.6, 3.8)	2.4 (1.6, 3.8)	2.3 (1.5, 4.2)	0.331
Myocardial infarct, n (%)				0.58
No	3763 (75.9)	3021 (76)	742 (75.2)	
Yes	1198 (24.1)	953 (24)	245 (24.8)	
Congestive heart failure, n (%)				< 0.001
No	2934 (59.1)	2407 (60.6)	527 (53.4)	
Yes	2027 (40.9)	1567 (39.4)	460 (46.6)	
Peripheral vascular disease, n (%)				0.256
No	4101 (82.7)	3273 (82.4)	828 (83.9)	
Yes	860 (17.3)	701 (17.6)	159 (16.1)	
Cerebrovascular disease, n (%)				< 0.001
No	4198 (84.6)	3405 (85.7)	793 (80.3)	
Yes	763 (15.4)	569 (14.3)	194 (19.7)	
Chronic pulmonary disease, n (%)				0.274
No	3439 (69.3)	2769 (69.7)	670 (67.9)	
Yes	1522 (30.7)	1205 (30.3)	317 (32.1)	
Liver disease, n (%)				< 0.001
No	4437 (89.4)	3619 (91.1)	818 (82.9)	
Yes	524 (10.6)	355 (8.9)	169 (17.1)	
Diabetes, n (%))				0.712
No	3597 (72.5)	2886 (72.6)	711 (72)	
Yes	1364 (27.5)	1088 (27.4)	276 (28)	
Renal disease, n (%)				< 0.001
No	3443 (69.4)	2803 (70.5)	640 (64.8)	
Yes	1518 (30.6)	1171 (29.5)	347 (35.2)	
Cancer, n (%)				< 0.001
No	4269 (86.1)	3481 (87.6)	788 (79.8)	
Yes	692 (13.9)	493 (12.4)	199 (20.2)	
Charlson comorbidity index	7.1 ± 2.4	6.9 ± 2.3	7.9 ± 2.6	< 0.001
APS III	63.4 ± 26.1	58.9 ± 23.9	81.7 ± 26.8	< 0.001
SAPS II	48.0 ± 13.2	46.4 ± 12.7	54.3 ± 13.3	< 0.001
OASIS	38.9 ± 9.3	37.6 ± 9.0	43.9 ± 8.7	< 0.001
SOFA	7.4 ± 2.9	7.1 ± 2.8	8.3 ± 3.4	< 0.001
Vasofree28, n (%)				< 0.001
No	1029 (20.7)	42 (1.1)	987 (100)	
Yes	3932 (79.3)	3932 (98.9)	0 (0)	
Ventfree28, n (%)				< 0.001
No	1028 (20.7)	41 (1)	987 (100)	
Yes	3933 (79.3)	3933 (99)	0 (0)	

Abbreviations: bpm = beats per minute; SBP = Systolic Blood Pressure; DBP = Diastolic Blood Pressure; bpm = breaths per minute; WBC = white blood cell; BUN = Blood Urea Nitrogen; APS III = Acute Physiology Score III; SAPS II = Simplified Acute Physiology Score II; OASIS = Outcome Prediction in the Intensive Care Unit: Simplified Acute Physiology Score; SOFA = Sequential Organ Failure Assessment; min = the lowest value; max = the highest value; Vasofree28 indicates that vasopressor agents were not used within 28 days after admission. Ventfree28 indicates that mechanical ventilation was not used within 28 days after admission.

### Identification of subpopulations using the JLCM

**Fig 1:**
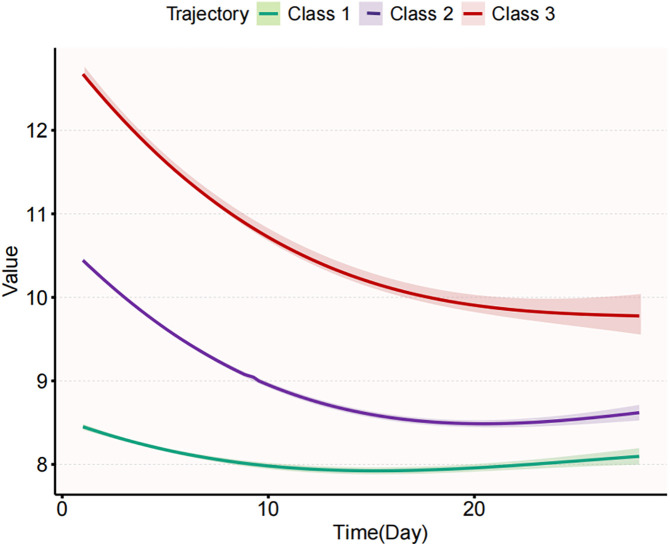
Trajectory curve. The figure illustrates the various trajectories of Hb values over survival time in all patients. The curves are divided into three classes (Class 1 to Class 3), each representing a distinct pattern of Hb variation. The vertical axis depicts the value of Hb, while the horizontal axis represents survival time (in days). Each class’s curve demonstrates the changes in Hb levels at different time points, accompanied by a shaded region indicating the estimated confidence interval.

The comparison of different potential trajectory models (2–5 classes) regarding goodness-of-fit and classification effectiveness is presented in Table 2. The 4-class model exhibits the best performance based on the Akaike Information Criterion (AIC) at 73305.1, the Bayesian Information Criterion (BIC) at 73422.3, and the Sample-Size Adjusted BIC (SBIC) at 73365.1. Additionally, it achieves a higher entropy value of 0.53897, which indicates a clearer classification. However, the proportion of Class 3 (9.63%) is significantly lower than that of the other classes (21.06%, 37.92%, and 30.84%). Although the 3-class model is slightly less effective than the 4-class model in terms of AIC (73398.0), BIC (73489.1), SBIC (73444.6), and entropy (0.50263), it still demonstrates clear classification. Furthermore, the proportions of patients in each category of the 3-class model are more balanced (23.63%, 23.48%, and 53.05%), and the probability indicators are higher, suggesting that this model can effectively differentiate between distinct trajectories. In conclusion, the 3-class model is identified as the optimal solution for trajectory analysis. The dynamic trajectories of the three model types are illustrated in Fig 1. The proportions of patients in the three classes are as follows: Class 1 (low-level maintenance, 23.63%); Class 2 (medium-level slow reduction, 53.05%); Class 3 (high-level rapid reduction, 23.48%).

**Table 2:** Metrics for determining the optimal trajectories.

Trajectory	Features					Proportion of Patients %)				
Class	Loglikelihood	AIC	BIC	SABIC	Entropy	Class1（%）	Class2（%）	Class3（%）	Class4（%）	Class5（%）
2	−36790.9	73601.8	73666.8	73635.1	0.69848	89.256	10.743	NA	NA	NA
3	−36685	73398	73489.1	73444.6	0.50263	23.463	53.053	23.483	NA	NA
4	−36634.5	73305.1	73422.3	73365.1	0.53897	21.608	37.915	9.6351	30.84	NA
5	−36601.4	73246.8	73390	73320.1	0.62447	19.935	31.203	31.263	12.618	4.9788

Abbreviations: AIC=akaike information criterion, BIC=bayesian information criteria, SABIC=sample-adjusted information criteria.

### Clinical characteristics of different Hb trajectories

The clinical characteristics of various Hb trajectory classes are presented in S2 Table. The results indicate significant differences among the classes in terms of blood pressure, respiratory rate, body temperature, blood oxygen levels, blood glucose, hematological parameters, and coagulation indices (p < 0.05). Notable variations in Hb changes were observed across different trajectories. Class 3 exhibited significantly higher Hb levels at all time points compared to the other classes, peaking at 13.4 g/dL on Day 1, but demonstrated a notable downward trend thereafter. Conversely, Class 1 consistently showed lower Hb levels at all time points, reaching a minimum of 7.0 g/dL on Day 5. Class 2 occupied an intermediate position, displaying a gradual and slow decline while remaining relatively stable within a certain range. Overall, Hb level changes in Class 3 were higher but declined rapidly, indicating a worsening degree of anemia, whereas Class 1 maintained a consistently low level, suggesting a persistent anemic state.

S3 Table presents the results of the univariate Cox regression analysis concerning 28-day mortality. Several clinical indicators—including age, blood gas parameters, biochemical markers, and underlying diseases—are significantly associated with the risk of mortality. Among the Hb trajectory classes, Class 1 (HR 1.64) and Class 3 (HR 1.28) notably elevate the risk of death, indicating that dynamic changes in Hb levels are crucial prognostic factors.

The analysis of covariates influencing Hb trajectory, informed by clinical significance and univariate Cox regression results, is presented in S4 Table. The screening outcomes identified several potential covariates associated with changes in Hb levels, including Hb value of the first day, respiratory rate, body temperature, and various blood gas parameters (such as chloride, base excess, and creatinine), as well as the Charlson comorbidity score, APACHE III, and OASIS score. These covariates demonstrated significant correlations with alterations in Hb trajectory (p < 0.05), with VIF values remaining within an acceptable range. As INR and PT exhibited multicollinearity, INR was ultimately chosen as the covariate for further analysis.

### Hazard Ratios from multivariable Cox model and Kaplan-Meier curves analysis

We conducted the Schoenfeld residual test to verify the assumption of proportional hazards in the Cox analysis. This assumption was validated for the endpoints of death from any cause, resulting in a p-value of 0.646.

In the multivariable Cox proportional hazards model presented in Table 3, the unadjusted model (Model 1) revealed that, compared to Class 2, Class 1 significantly increased the risk of mortality (HR 1.64, p < 0.001), while Class 3 also exhibited a notable increase in risk (HR 1.28, p = 0.001). Model 2 adjusted for age and sex, and Model 3 further incorporated laboratory indices based on Model 2. Model 4 demonstrated that after adjusting for all covariates: age, respiratory rate mean, temperature mean, anion gap maximum, bicarbonate minimum, blood urea nitrogen maximum, creatinine maximum, international normalized ratio maximum, SAPS II, OASIS, and the Charlson comorbidity index, the Hb trajectory remained an independent prognostic indicator: Class 1 (HR = 1.38, 95% CI = 1.18–1.62, p < 0.001) and Class 3 (HR = 1.21, 95% CI = 1.03–1.42, p = 0.020), as illustrated in Table 3.

**Table 3:** Relationship between different Hb Classes and 28-day morality in different models.

Variable	Model1		Model2		Model3		Model4	
	HR (95%CI)	p	HR (95%CI)	p	HR (95%CI)	p	HR (95%CI)	p
class2	1(Ref)		1(Ref)		1(Ref)		1(Ref)	
class1	1.64 (1.42 ~ 1.91)	<0.001	1.67 (1.44 ~ 1.94)	<0.001	1.31 (1.12 ~ 1.53)	0.001	1.38 (1.18 ~ 1.62)	<0.001
class3	1.28 (1.1 ~ 1.5)	0.001	1.3 (1.11 ~ 1.52)	0.001	1.16 (0.99 ~ 1.37)	0.066	1.21 (1.03 ~ 1.42)	0.02

Model1: crude model.

Model2: adjusted for age, gender.

Model3: adjusted for age, sex, Resp rate-mean, Temperature-mean, anion gap-max, Bicarbonate-min, Bun-max, Creatinine-max, INR-max.

Model4: adjusted for age, sex, Resp rate-mean, Temperature-mean, anion gap-max, Bicarbonate-min, Bun-max, Creatinine-max, INR-max, Chronic pulmonary disease, SAPSII, OASIS.

**Fig 2:**
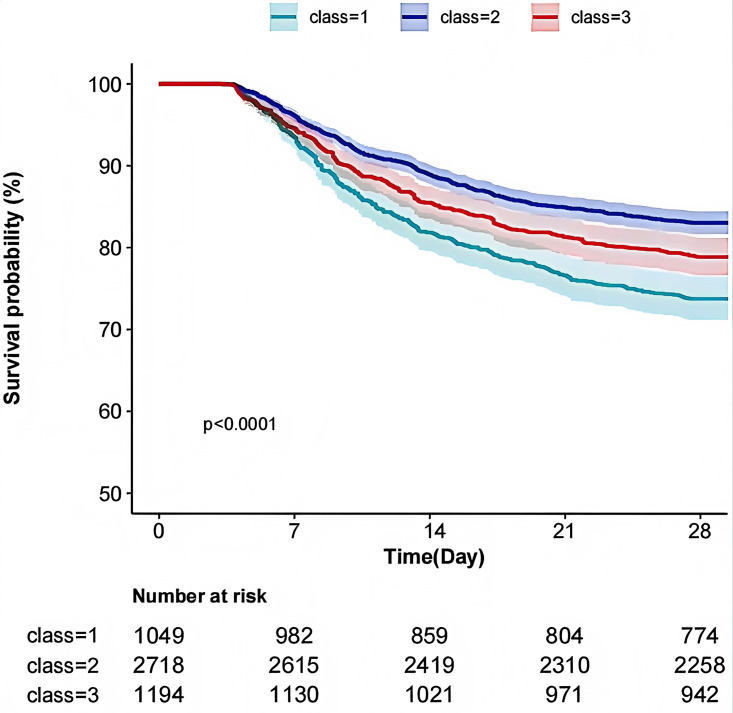
KM curves for different trajectories. The figure illustrates the survival probabilities of three different Hb change trajectories. The vertical axis represents survival probability (%), while the horizontal axis indicates the trajectory classes. The curves of different colors correspond to distinct trajectory classes (Trajectory 1 to Trajectory 3). The graph reveals significant differences in survival probabilities over time, with class 1 (in green) showing the lowest survival probability, falling to about 75% within 28 days. Class 2(in purple)showing the highest survival probability followed by Class 3(in red).The p-value < 0.0001 indicates that the differences in survival rates among the trajectories are statistically significant. The table below lists the number at risk for each trajectory at different time points(in days).

The Kaplan-Meier curves derived from the multivariate analysis indicated that varying Hb trajectories (Classes 1, 2, and 3) were significantly correlated with the 28-day survival probability (p < 0.001). Specifically, the category exhibiting the lowest Hb level (class 1) demonstrated the poorest survival rate, whereas Class 3 exhibited a survival rate higher than that of Class 1 but lower than that of Class 2, as illustrated in Fig 2.

### Sensitivity analysis

A subgroup analysis was conducted as part of a sensitivity analysis. S2 Fig illustrates the subgroup classifications and event occurrences among all patients, assessing the impact of various factors such as sex, age, congestive heart failure, cerebrovascular disease, diabetes, liver disease, kidney disease, cancer, and the Charlson comorbidity index, SAPSII, and OASIS (with Age, Charlson comorbidity index, SAPSII, and OASIS categorized based on the median) on mortality risk, measured as the hazard ratio (HR). In all subgroup analyses for each category, the p-values were greater than 0.05, confirming the robustness of the results.

To mitigate the potential effects of unmeasured confounders, we conducted an unmeasured confounding E-value analysis. The E-value for the point estimate was 1.249, with a lower confidence interval of 1.121 and an upper confidence interval of 1.395. These relatively large E-values indicate the robustness of our findings, as illustrated in S3 Fig.

**Fig 3:**
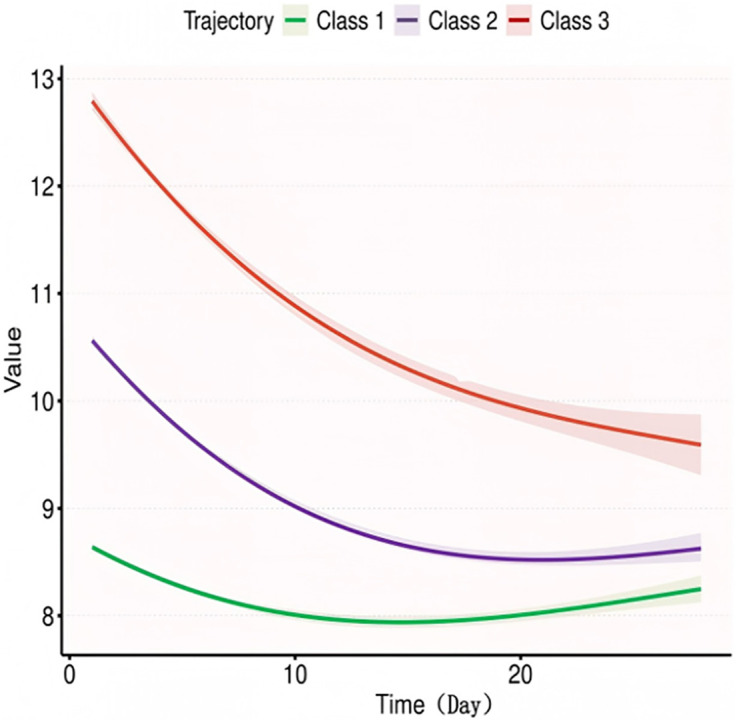
Trajectory curve for Male.

**Fig 4:**
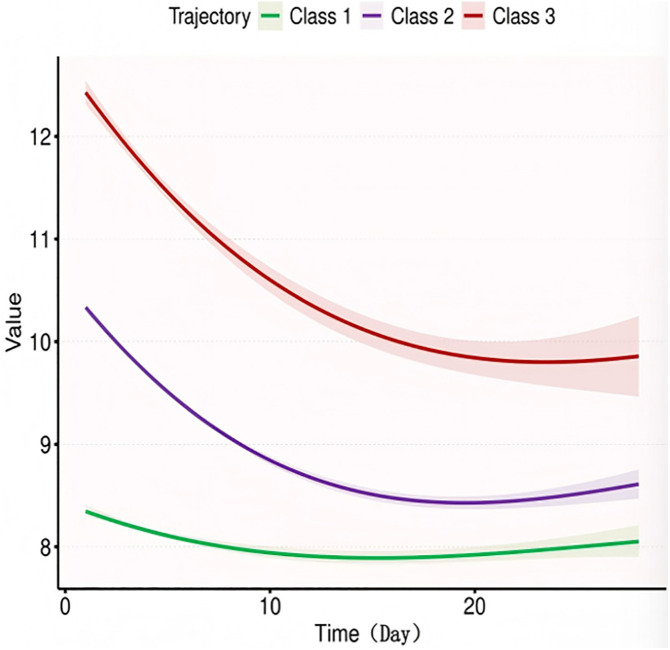
Trajectory curve for Female.

**Fig 5:**
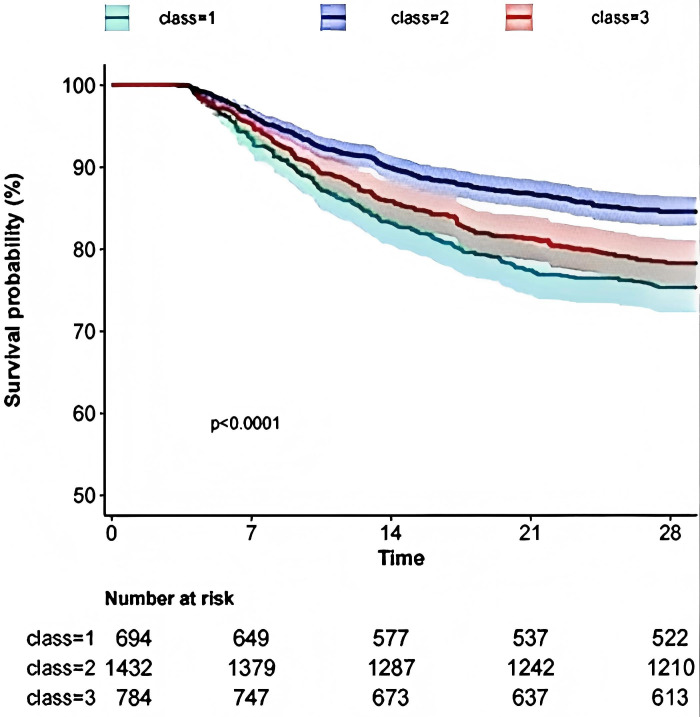
KM curves for Male group.

**Fig 6:**
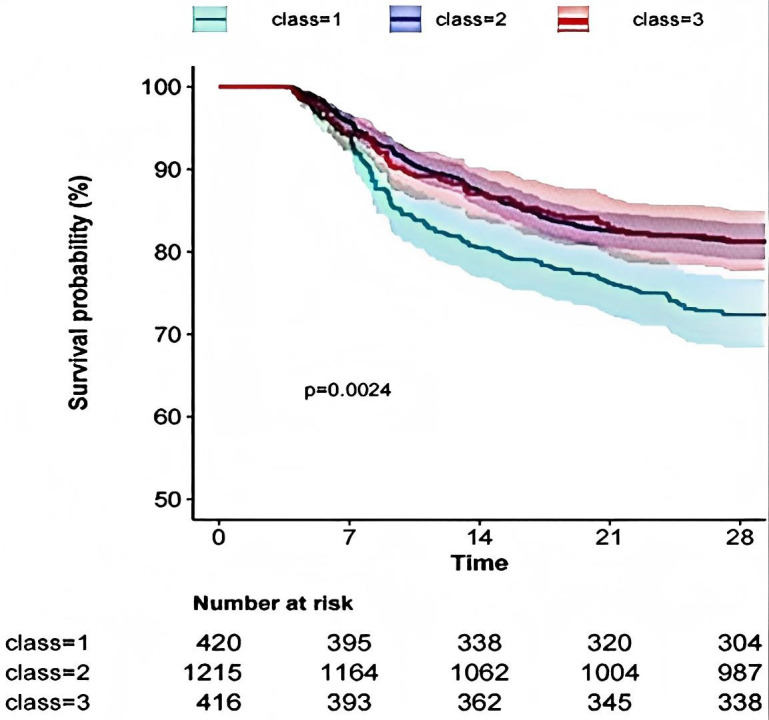
KM curves for Female group.

Considering the differences in Hb reference values between genders, we conducted trajectory analyses separately for males and females, as illustrated in Figs 3 and 4. Overall, males exhibited slightly higher Hb levels at various time points compared to females; however, the trajectories remained consistent across genders. In terms of Hb values at different time points, significant differences were observed between the survival and death groups for males on Day 7, Day 14, and in the minimum Hb values. For females, differences were noted between the survival and death groups on Day 1, Day 5, Day 7, Day 14, as well as in the maximum, minimum, and average values, as detailed in Table 4. The K-M curves derived from the multivariate analysis indicated that varying Hb trajectories (Classes 1, 2, and 3) were significantly correlated with the 28-day survival probability in both male and female (both p < 0.05), as depicted in, Figs 5 and 6.

**Table 4:** Hb value Characteristics at different time points of different gender.

Gender	Male				Female			
Variables	Total (n = 2910)	Survival group (n = 2345)	Death group (n = 565)	p	Total (n = 2051)	Survival group (n = 1629)	Death group (n = 422)	p
Hb Day1	11.0 ± 2.0	11.1 ± 1.9	11.0 ± 2.2	0.296	10.7 ± 1.8	10.7 ± 1.7	10.4 ± 1.8	0.001
Hb Day3	9.8 ± 1.6	9.8 ± 1.6	9.9 ± 1.8	0.194	9.5 ± 1.5	9.6 ± 1.4	9.5 ± 1.5	0.254
Hb Day5	9.8 ± 1.6	9.9 ± 1.6	9.8 ± 1.7	0.155	9.6 ± 1.5	9.6 ± 1.5	9.4 ± 1.5	0.001
Hb Day7	9.8 ± 1.6	9.8 ± 1.6	9.6 ± 1.7	0.038	9.5 ± 1.5	9.5 ± 1.5	9.2 ± 1.5	0.001
Hb Day14	9.2 ± 1.5	9.3 ± 1.5	9.1 ± 1.6	0.041	9.0 ± 1.3	9.0 ± 1.3	8.7 ± 1.2	0.002
Hb Day21	8.8 ± 1.3	8.8 ± 1.3	8.5 ± 1.3	0.033	8.7 ± 1.1	8.7 ± 1.1	8.4 ± 1.0	0.098
Hb Day28	8.7 ± 1.1	8.7 ± 1.1	8.4 ± 0.8	0.414	8.6 ± 1.1	8.6 ± 1.1	7.8 ± 1.0	0.087
Hb-max	11.4 ± 1.8	11.4 ± 1.7	11.3 ± 2.1	0.333	11.0 ± 1.6	11.1 ± 1.6	10.8 ± 1.7	< 0.001
Hb-min	8.5 ± 1.7	8.5 ± 1.6	8.3 ± 1.9	0.027	8.1 ± 1.5	8.1 ± 1.5	8.0 ± 1.5	0.074
Hb-mean	9.8 ± 1.5	9.8 ± 1.4	9.7 ± 1.7	0.24	9.5 ± 1.3	9.5 ± 1.3	9.3 ± 1.4	0.012
Class n (%)				< 0.001				< 0.001
1	694 (23.8)	522 (22.3)	172 (30.4)		420 (20.5)	304 (18.7)	116 (27.5)	
2	1432 (49.2)	1210 (51.6)	222 (39.3)		1215 (59.2)	987 (60.6)	228 (54)	
3	784 (26.9)	613 (26.1)	171 (30.3)		416 (20.3)	338 (20.7)	78 (18.5)	

The multivariate regression model indicated that the dynamic trajectory of Hb in males had a higher predictive value for 28-day survival compared to females (Tables 5 and 6). In the unadjusted model for males, Class 1 was associated with a significantly increased risk of mortality compared to Class 2 (HR 1.70, p < 0.001), while Class 3 also exhibited an upward trend (HR 1.46, p < 0.001). After adjustments (Models 2–4), the risk in the Class 1 group remained significant (HR 1.21–1.67, p < 0.05). Furthermore, the risk for females in the HbClass 1 group was also significantly elevated (HR 1.38–1.59, p < 0.01), although no significant difference was observed in the risk for Class 3. These findings suggest that persistently low Hblevels serve as an independent predictor of mortality across both genders.

**Table 5:** Metrics for the trajectories for Male and Female.

	Trajectory	Features					Proportion of Patients (%)				
Gender	class	Loglikelihood	AIC	BIC	SABIC	Entropy	Class1（%）	Class2（%）	Class3（%）	Class4（%）	Class5（%）
Male	2	−21768.2	43556.44	43616.20	43584.42	0.333568	68.10996	31.89003	NA	NA	NA
	3	−21699.0	43426.08	43509.75	43465.26	0.497784	23.84879	26.94158	49.20962	NA	NA
	4	−21667.4	43370.94	43478.50	43421.31	0.535564	18.28178	32.74914	15.22336	33.74570	NA
	5	−21644.9	43333.97	43465.44	43395.54	0.633019	17.18213	31.27147	33.19587	12.19931	6.151202
Female	2	−14581.9	29193.89	29278.28	29230.62	0.686660	86.83568	13.16431	NA	NA	NA
	3	−14511.7	29063.47	29175.99	29112.45	0.541274	20.47781	59.23939	20.28278	NA	NA
	4	−14484.2	29018.55	29159.20	29079.78	0.536154	21.01413	39.73671	33.25207	5.997074	NA
	5	−14462.7	28985.49	29154.28	29058.96	0.616793	4.973183	58.70307	25.25597	3.949293	7.118478

Abbreviations: AIC akaike information criterion, BIC bayesian information criteria, SABIC sample-adjusted information criteria.

**Table 6:** Relationship between different Hb Classes and 28-day mortality in different models and Genders.

Gender	Variable	Model1		Model2		Model3		Model4	
		HR (95%CI)	P	HR (95%CI)	P	HR (95%CI)	P	HR (95%CI)	P
Male	class2	1(Ref)		1(Ref)		1(Ref)		1(Ref)	
	class1	1.7 (1.39 ~ 2.07)	<0.001	1.7 (1.39 ~ 2.07)	<0.001	1.24 (1.01 ~ 1.53)	0.042	1.21 (0.98 ~ 1.5)	0.071
	class3	1.46 (1.19 ~ 1.78)	<0.001	1.48 (1.21 ~ 1.81)	<0.001	1.22 (0.99 ~ 1.51)	0.058	1.4 (1.13 ~ 1.73)	0.002
Female	class2	1(Ref)		1(Ref)		1(Ref)		1(Ref)	
	class1	1.56 (1.25 ~ 1.95)	<0.001	1.59 (1.27 ~ 1.99)	<0.001	1.38 (1.09 ~ 1.74)	0.007	1.43 (1.13 ~ 1.81)	0.003
	class3	1 (0.78 ~ 1.3)	0.974	0.99 (0.76 ~ 1.28)	0.923	0.99 (0.75 ~ 1.29)	0.922	1.04 (0.8 ~ 1.37)	0.752

Model1: crude model.

Model2: adjusted for age, gender.

Model3: adjusted for age, sex, Resp rate-mean, Temperature-mean, Anion gap-max, Bicarbonate-min, Bun-max, Creatinine-max, INR-max.

Model4: adjusted for age, sex, Resp rate-mean, Temperature-mean, anion gap-max, Bicarbonate-min, Bun-max, Creatinine-max, INR-max, Chronic pulmonary disease, SAPSII, OASIS.

## Discussion

To our knowledge, this study is the first to explore the relationship between dynamic Hb value trajectories and 28-day mortality risk in elderly sepsis patients. The Cox model demonstrates that dynamic trajectories can indirectly reflect treatment effects and changes in patients’ pathophysiology compared to single Hb measurements at specific time points, thereby providing a more accurate prognostic prediction. This study indicates that trajectories of Hblevels characterized by persistently low values or a rapid decrease from near-normal values are associated with an increased risk of 28-day mortality compared to trajectories that decline slowly around the median value. While Huang et al. pioneered trajectory phenotyping in sepsis, this study establishes its context-specific clinical utility in high-risk geriatric populations [11]. We further observed greater physiological tolerance to anemia severity among elderly females compared to males, a finding that addresses critical knowledge gaps in personalized management for aging demographics.

For a long time, Hb levels have been considered associated with disease prognosis. An initial Hblevel of 80 g/L or lower within the first 48 hours of ICU admission is recognized as a predictive indicator of one-year mortality in severe sepsis, underscoring the significance of early Hb measurements in sepsis prognosis [27,28]. Insufficient Hbcan lead to inadequate tissue oxygenation, while an inappropriate increase in Hb may indicate a hypercoagulable state or even thrombosis [29]. Research has demonstrated a non-linear relationship between Hb levels and various diseases, including renal disease and the mortality rate during acute exacerbations of COPD [29]. Recent studies indicate that admission Hb levels also exhibit a non-linear relationship with 30-day mortality in sepsis patients. Specifically, with a threshold of 7.2 g/dL, each 1 g/dL increase in Hb is associated with an approximately 13% reduction in the odds ratio (OR) for 30-day mortality. This finding suggests that maintaining Hb levels above 7.2 g/dL may reduce the risk of death in patients with sepsis [30].Although decreased Hb levels may indeed indicate a more critically ill patient, the Hb level itself is not the core issue. Rather, the changes in Hb levels reflect a disturbance in the inflammatory state, which may lead to adverse outcomes. Hb serves as a surrogate marker for inflammation and may indicate complications such as macrophage activation syndrome or other similar conditions. Mounting evidence underscores hemoglobin’s (Hb) immunoregulatory role. Hb levels modulate immune cell activity and inflammatory responses, positioning them as potential surrogate markers for immune status across diverse pathologies. Within COVID-19, lower Hb correlates with elevated inflammatory biomarkers (e.g., CRP, IL-6) and increased disease severity [31]. Among patients with systemic lupus erythematosus (SLE), Hb levels inversely associate with disease activity and inflammation, underscoring its immunomodulatory potential [32]. In oncology, Hb serves as a composite prognostic biomarker (e.g., HB-CEA) for predicting disease progression and immune response [33]. The underlying mechanisms entail Hb’s critical function in oxygen delivery for immune cell metabolism and its direct interaction with cytokines (e.g., IL-6, IL-8) [34]. Nevertheless, confounding factors—including age, sex, and comorbidities—complicate Hb’s interpretation as a universal immune marker, necessitating further investigation to address these limitations. Monitoring Hb trajectories offers a practical clinical tool: not to target Hb therapeutically in isolation, but to identify high-risk patients warranting intensified investigation for underlying inflammation, infection, or organ dysfunction.

The correlation between Hb levels and 28-day mortality in elderly patients with sepsis represents an innovative study, as this demographic often presents complex pathological conditions, including anemia, malnutrition, decline in cardiopulmonary function, and other comorbidities, which complicate prognostic analysis. The average Hb (concentration in the elderly population tends to gradually decrease with age [35]. Furthermore, the higher prevalence of anemia among older adults is frequently linked to various comorbidities, such as chronic kidney disease, cardiovascular disorders, and malnutrition. Additionally, elderly individuals with anemia are at an increased risk for adverse clinical outcomes, including elevated hospitalization rates, frailty, cognitive decline, and higher mortality [36]. Although age was not included as a covariate in the trajectory modeling, sensitivity analyses revealed no significant differences in age distribution across trajectory groups (p _for interaction_ = 0.241 in age subgroup analysis)(S2 Fig). However, unmeasured age-related physiological changes (e.g., bone marrow hematopoietic) may still influence trajectory classification, constituting a potential source of residual confounding. This study reveals that Hb levels in elderly patients with sepsis are comparatively lower than established normal reference values, aligning with the recognized phenomenon that critically ill elderly patients typically exhibit diminished Hb levels. Notably, approximately 40% of patients experience a reduction in Hb levels to 10 g/dL following hospitalization [37].

The trend of Hb changes during the treatment process may indicate alterations in clinical conditions. For instance, the presence of persistently worsening anemia in a patient may suggest a deterioration in clinical status, potentially necessitating further blood transfusions to correct the anemia or enhance supportive therapy [38]. This study is distinguished by its focus on the association between Hb trajectory and 28-day mortality. We collected Hb data for patients upon admission and at days 1, 3, 5, 7, 14, 21, and 28 for trajectory analysis, which enhanced predictive capability. Furthermore, the results of the subgroup analysis confirmed the stability of the findings. Upon reclassifying and analyzing the trajectories by gender, we found that the trajectory classifications were largely consistent between males and females. However, the Hb values at various time points were higher in males than in females, and the predictive power of the third category’s accumulation for early mortality risk was stronger in males than in females. Additionally, we found that female, compared to male, exhibit a greater tolerance for rapid decreases in Hb levels and a relatively lower risk of adverse outcomes.

Early studies indicate gender differences in anemia tolerance. Males exhibit higher mortality rates at lower nadir Hb levels, particularly at 6.0 g/dL or less, whereas females do not show a corresponding risk increase. Adjusted for confounders, inpatient mortality is significantly higher in males under similar conditions, suggesting females better tolerate lower Hb levels [39]. Consistent with prior studies, our findings confirm greater tolerance to anemia among elderly women compared to men. This is evidenced by significantly higher inpatient mortality rates in males at lower Hb levels (e.g., ≤ 6.0 g/dL) even after confounder adjustment—a risk elevation not observed in females [39]. This physiological advantage in women likely stems from combined long-term adaptation and distinct inflammatory regulation mechanisms. First, lifelong adaptation to relatively lower Hb levels through physiological blood loss (e.g., menstruation, pregnancy) may enhance functional resilience during anemic episodes [40]. Second, a key mechanistic difference involves iron metabolism under inflammatory conditions: men exhibit significantly higher hepcidin levels (a hormone inhibiting iron availability), whereas lower hepcidin in women facilitates more effective iron utilization and erythropoiesis, thereby mitigating anaemia severity [41]. Collectively, these factors confer greater tolerance to Hb decline in women.

Our study possesses several strengths. Firstly, it emphasizes the 28-day mortality rates among elderly sepsis patients, a demographic typically characterized by more severe disease states, a higher burden of comorbidities, and an increased need for clinical intervention. Secondly, this research leverages public databases and employs repeated Hb measurements from the first month of ICU hospitalization to establish dynamic trajectory patterns. Furthermore, it conducts proportional hazards testing to explore potential time cutoff points within the study duration. Finally, the JLCM method serves as a form of unsupervised trajectory analysis, facilitating the identification of valuable insights. Additionally, we conducted a series of sensitivity analyses, including the Schoenfeld residual test, subgroup analysis, and E-value testing, to ensure the robustness of our study results.

This study has several inherent limitations. The analytical data were obtained from a public database of a single research institution, which restricted the extraction and joint analysis of imaging features, treatment protocols, and nursing measures, as well as the monitoring of additional therapeutic outcomes and lifestyle such as smoking status, a known modifier of Hb metabolism and sepsis pathophysiology was not available in our data source. The final cohort comprised 4,961 patients with 987 mortality events, with an Events Per Variable (EPV) ratio of approximately 90 (11 covariates in the fully adjusted model). This far exceeds the recommended EPV threshold of 10, ensuring robust statistical power for our Cox regression analysis [22]. However, it is important to note that the sensitivity of p-values to sample size, combined with the misinterpretation of their meaning, has contributed to the reproducibility crisis in scientific research. While larger sample sizes can improve the power to detect small effects, they also increase the likelihood of identifying statistically significant but clinically insignificant results [42]. The E-value is utilized as a method for sensitivity analyses to clarify the extent to which the study results may be influenced by unmeasured confounding factors. Nevertheless, the relatively large E-values bolster the reliability of the findings.

## Conclusion

In summary, dynamic analysis of Hb trajectory provides critical prognostic information regarding mortality in elderly patients with sepsis. Effective monitoring and management of Hb may be a key strategy for improving clinical outcomes in this high-risk group, addressing the dual challenges of sepsis and anemia. Further research aimed at optimizing treatment approaches based on these dynamic trajectories could ultimately enhance clinical prognoses for this specific population.

## Supporting information

S1 Fig Flowchart of study population. (TIF)

S2 Fig Forest plot of subgroup analyses. (TIF)

S3 Fig E value bias plot. (TIF)

S1 Table Missing data. (DOC)

S2 Table Clinical Characteristics of Different Hemoglobin Trajectories. (DOC)

S3 Table Univariate Cox Regression Analysis Results for 28-Day Mortality. (DOC)

S4 Table The results of covariate selection. (DOC)
