# Supplementary figures and images for: Association between hemoglobin dynamic trajectories and 28-day mortality in elderly patients with sepsis: A retrospective cohort study using the MIMIC-IV database
Source: PLoS One. 2026 May 4;21(5):e0327443. doi: 10.1371/journal.pone.0327443 (PMC13138669; doi:10.1371/journal.pone.0327443)

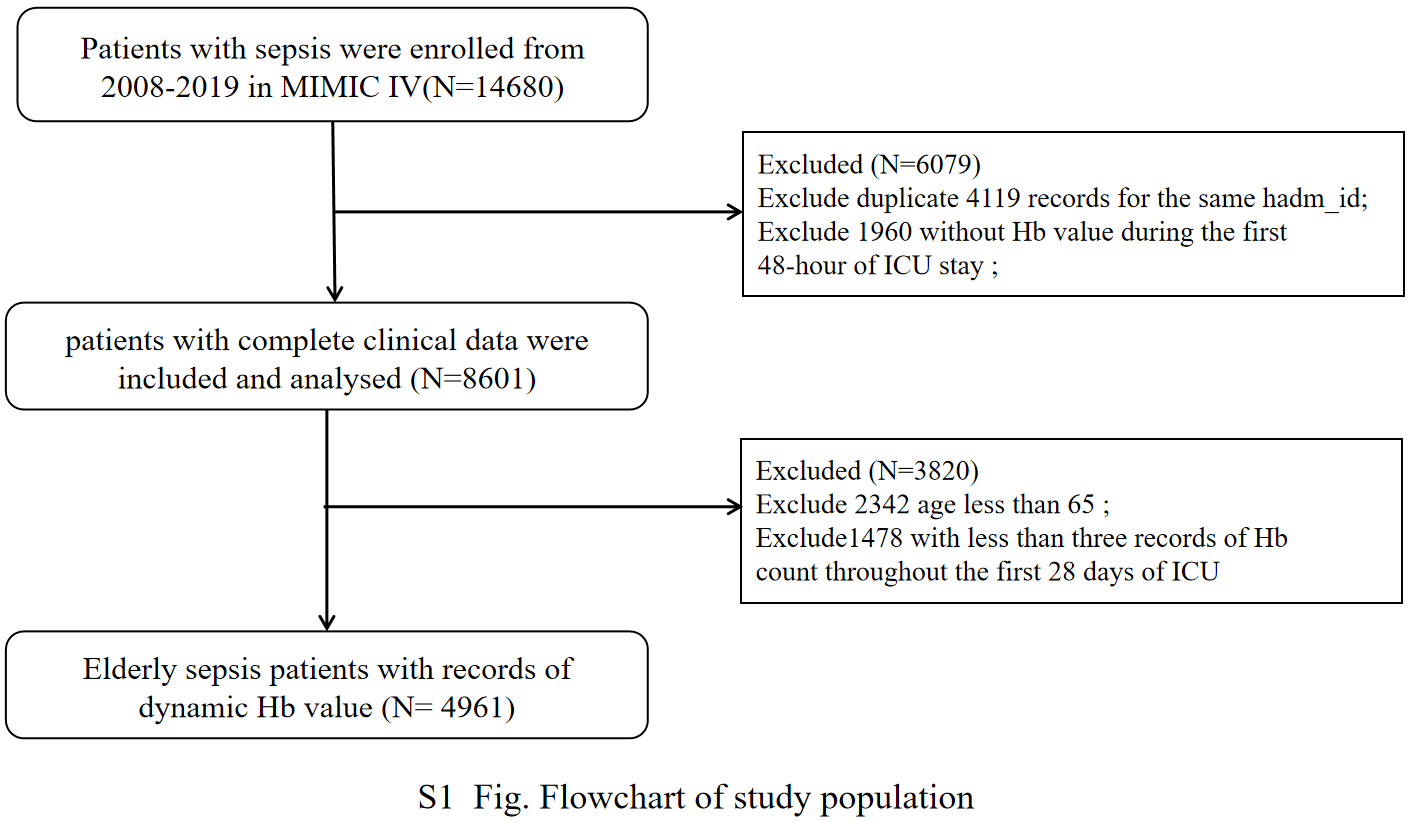

Supplement: S1 Fig — (TIF) [file pone.0327443.s001.tif]

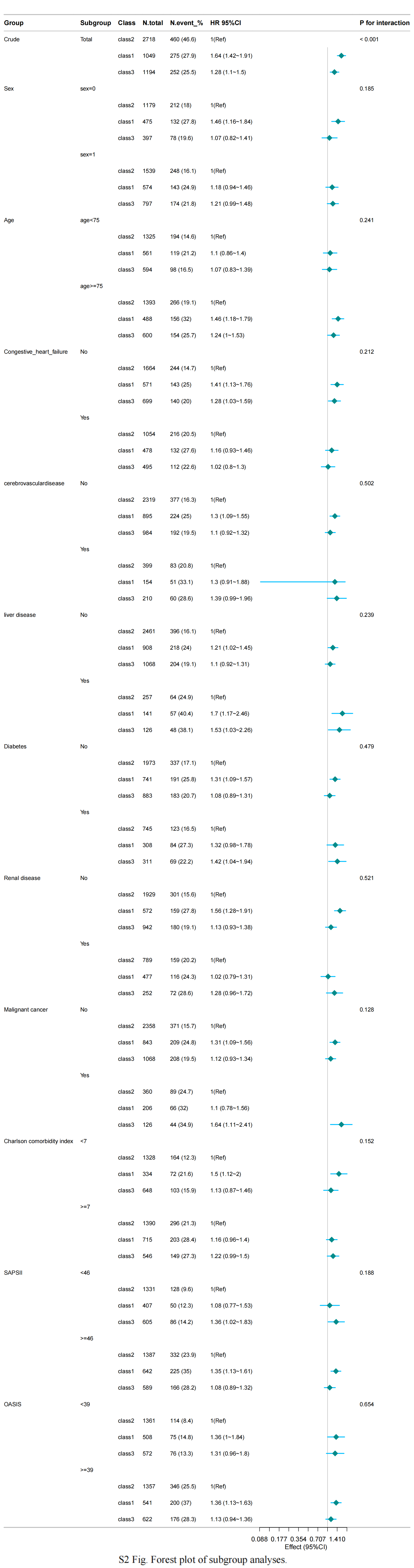

Supplement: S2 Fig — (TIF) [file pone.0327443.s002.tif]

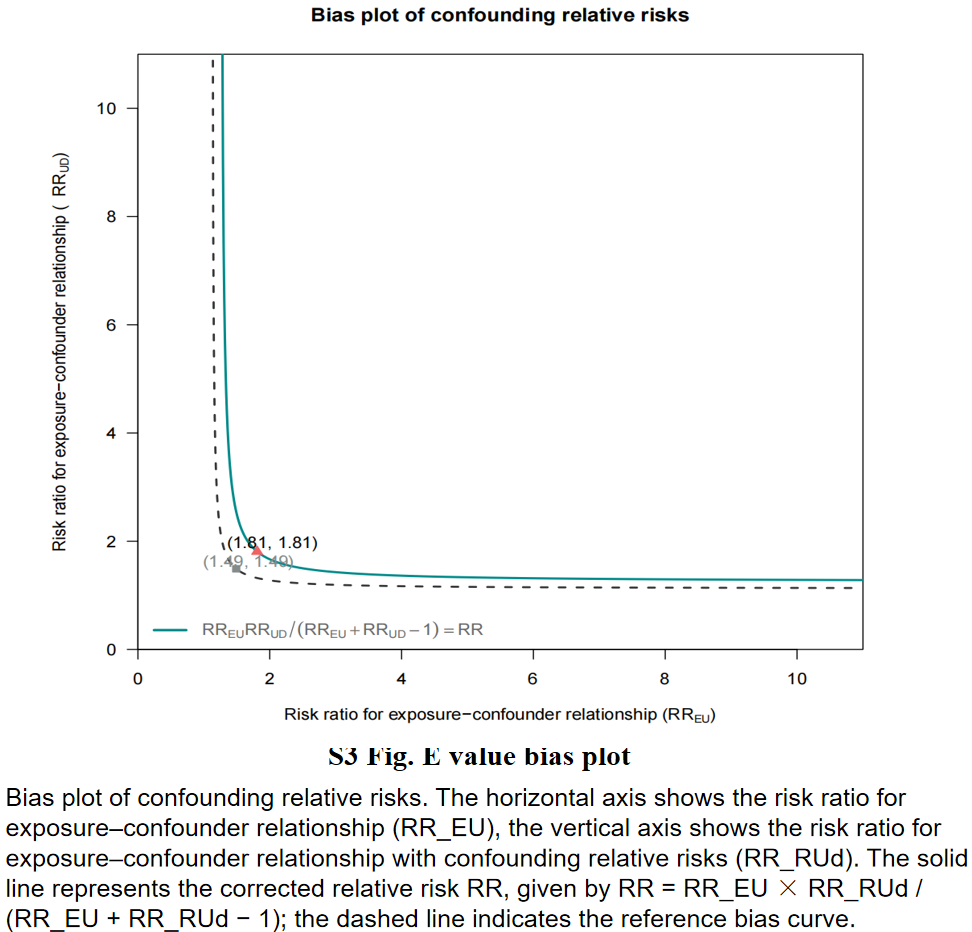

Supplement: S3 Fig — (TIF) [file pone.0327443.s003.tif]
