# Supplementary material for: Association between hemoglobin dynamic trajectories and 28-day mortality in elderly patients with sepsis: A retrospective cohort study using the MIMIC-IV database
Source: PLoS One. 2026 May 4;21(5):e0327443. doi: 10.1371/journal.pone.0327443 (PMC13138669; doi:10.1371/journal.pone.0327443)
Supplement: S1 Table — (DOC) [file pone.0327443.s004.doc]

| **S1 Table. Missing data.** | | |
| --- | --- | --- |
| Variable | Miss.num | Miss.percentage% |
| CRP | 4237 | 85.41% |
| Albumin | 3228 | 65.07% |
| Hb28 | 629 | 12.68% |
| Calcium-min | 575 | 11.59% |
| Temperature-mean | 414 | 8.35% |
| Hb21 | 356 | 7.18% |
| Hb7 | 244 | 4.92% |
| APTT-max | 171 | 3.45% |
| INR-max | 153 | 3.08% |
| PT-max | 153 | 3.08% |
| Hb14 | 149 | 3.00% |
| Hb3 | 135 | 2.72% |
| Hb5 | 133 | 2.68% |
| DBP-mean | 34 | 0.69% |
| SBP-mean | 33 | 0.67% |
| Aniongap_max | 12 | 0.24% |
| Potassium_max | 9 | 0.18% |
| Heart rate-mean | 5 | 0.10% |
| Resp rate-mean | 5 | 0.10% |
| Sodium-min | 5 | 0.10% |
| SOFA | 5 | 0.10% |
| Spo2-mean | 5 | 0.10% |
| Glucose-mean | 4 | 0.08% |
| Platelets-min | 3 | 0.06% |
| WBC-max | 3 | 0.06% |
| Bicarbonate-min | 2 | 0.04% |
| BUN-max | 2 | 0.04% |
| Chloride-min | 2 | 0.04% |
| Creatinine-max | 2 | 0.04% |
