# Supplementary material for: Association between hemoglobin dynamic trajectories and 28-day mortality in elderly patients with sepsis: A retrospective cohort study using the MIMIC-IV database
Source: PLoS One. 2026 May 4;21(5):e0327443. doi: 10.1371/journal.pone.0327443 (PMC13138669; doi:10.1371/journal.pone.0327443)
Supplement: S2 Table — (DOC) [file pone.0327443.s005.doc]

| **S2 Table. Clinical Characteristics of Different Hemoglobin Trajectories.** | | | | | |
| --- | --- | --- | --- | --- | --- |
| **Variables** | **Total (n = 4961)** | **2 (n = 2718)** | **1 (n = 1049)** | **3 (n = 1194)** | **p** |
| Age | 77.2 ± 7.7 | 76.4 ± 7.7 | 77.3 ± 7.6 | 77.8 ± 7.8 | 0.015 |
| Heart rate-mean (bpm) | 84.8 ± 15.1 | 84.5 ± 14.1 | 84.7 ± 15.8 | 85.5 ± 16.6 | 0.15 |
| SBP-mean (mmHg) | 113.8 ± 13.0 | 112.8 ± 12.3 | 115.0 ± 13.7 | 114.7 ± 14.0 | < 0.001 |
| DBP-mean (mmHg) | 57.8 ± 9.0 | 56.6 ± 8.3 | 56.7 ± 9.0 | 61.6 ± 9.5 | < 0.001 |
| Resp rate-mean(bpm) | 19.7 ± 3.8 | 19.3 ± 3.8 | 20.3 ± 3.8 | 20.0 ± 3.9 | < 0.001 |
| Temperature-mean(℃) | 36.8 ± 0.6 | 36.8 ± 0.6 | 36.9 ± 0.6 | 36.9 ± 0.6 | 0.004 |
| SpO2-mean (%) | 97.2 ± 2.0 | 97.4 ± 1.9 | 97.2 ± 1.9 | 96.7 ± 2.0 | < 0.001 |
| Glucose-mean (mg/dL) | 137.0 (120.6, 166.4) | 135.1 (120.5, 161.7) | 139.8 (119.2, 174.4) | 139.2 (122.0, 169.7) | < 0.001 |
| Platelets-min (×10^9^/L) | 146.0 (102.0, 206.8) | 140.0 (100.0, 199.0) | 156.0 (96.5, 237.5) | 154.0 (109.0, 204.0) | < 0.001 |
| WBC-max (×10^9^/L) | 15.0 (11.0, 20.2) | 15.1 (11.2, 20.2) | 14.8 (10.3, 20.7) | 15.2 (11.2, 19.7) | 0.264 |
| Anion gap-max (mmol/L) | 16.0 (13.0, 19.0) | 15.0 (13.0, 19.0) | 17.0 (14.0, 21.0) | 16.0 (14.0, 19.0) | < 0.001 |
| Bicarbonate-min (mmol/L) | 20.3 ± 5.0 | 20.4 ± 4.8 | 19.7 ± 5.7 | 20.7 ± 4.9 | < 0.001 |
| BUN-max (mg/dL) | 27.0 (19.0, 45.0) | 26.0 (18.0, 41.0) | 39.0 (23.0, 62.0) | 26.0 (18.0, 39.0) | < 0.001 |
| Calcium-min (mg/dL) | 7.9 ± 0.9 | 7.9 ± 0.9 | 7.9 ± 0.9 | 7.9 ± 0.9 | 0.744 |
| Chloride-min (mmol/L) | 102.8 ± 6.6 | 103.4 ± 6.3 | 101.7 ± 7.1 | 102.4 ± 6.5 | < 0.001 |
| Creatinine-max (mg/dL) | 1.3 (0.9, 2.2) | 1.3 (0.9, 2.0) | 1.8 (1.1, 3.1) | 1.2 (0.9, 1.8) | < 0.001 |
| Sodium-min (mmol/L) | 137.0 ± 5.2 | 137.1 ± 4.9 | 136.8 ± 5.7 | 137.2 ± 5.5 | 0.205 |
| Potassium-max (mmol/L) | 4.8 ± 0.8 | 4.7 ± 0.8 | 4.8 ± 0.9 | 4.7 ± 0.9 | < 0.001 |
| INR-max (s) | 1.4 (1.2, 1.7) | 1.4 (1.2, 1.7) | 1.4 (1.2, 1.8) | 1.4 (1.2, 1.7) | 0.004 |
| PT-max (s) | 15.6 (13.6, 18.6) | 15.8 (13.8, 18.5) | 15.5 (13.6, 19.3) | 15.3 (13.3, 18.7) | 0.003 |
| APTT-max (s) | 35.3 (29.7, 49.8) | 35.6 (30.1, 48.9) | 33.9 (29.0, 46.7) | 35.9 (29.6, 56.3) | 0.002 |
| Lactate-max (mmol/L) | 2.4 (1.6, 3.8) | 2.5 (1.6, 4.0) | 1.9 (1.3, 3.4) | 2.4 (1.7, 3.8) | < 0.001 |
| Myocardial infarct, n (%) |  |  |  |  | 0.103 |
| No | 3763 (75.9) | 2066 (76) | 815 (77.7) | 882 (73.9) |  |
| Yes | 1198 (24.1) | 652 (24) | 234 (22.3) | 312 (26.1) |  |
| Congestive heart failure, n (%) |  |  |  |  | < 0.001 |
| No | 2934 (59.1) | 1664 (61.2) | 571 (54.4) | 699 (58.5) |  |
| Yes | 2027 (40.9) | 1054 (38.8) | 478 (45.6) | 495 (41.5) |  |
| Peripheral vascular disease, n (%) |  |  |  |  | < 0.001 |
| No | 4101 (82.7) | 2186 (80.4) | 889 (84.7) | 1026 (85.9) |  |
| Yes | 860 (17.3) | 532 (19.6) | 160 (15.3) | 168 (14.1) |  |
| Cerebrovascular disease, n (%) |  |  |  |  | 0.053 |
| No | 4198 (84.6) | 2319 (85.3) | 895 (85.3) | 984 (82.4) |  |
| Yes | 763 (15.4) | 399 (14.7) | 154 (14.7) | 210 (17.6) |  |
| Chronic pulmonary disease, n (%) |  |  |  |  | 0.968 |
| No | 3439 (69.3) | 1884 (69.3) | 730 (69.6) | 825 (69.1) |  |
| Yes | 1522 (30.7) | 834 (30.7) | 319 (30.4) | 369 (30.9) |  |
| Liver disease, n (%) |  |  |  |  | 0.002 |
| No | 4437 (89.4) | 2461 (90.5) | 908 (86.6) | 1068 (89.4) |  |
| Yes | 524 (10.6) | 257 (9.5) | 141 (13.4) | 126 (10.6) |  |
| Diabetes, n (%) |  |  |  |  | 0.212 |
| No | 3597 (72.5) | 1973 (72.6) | 741 (70.6) | 883 (74) |  |
| Yes | 1364 (27.5) | 745 (27.4) | 308 (29.4) | 311 (26) |  |
| Renal disease, n (%) |  |  |  |  | < 0.001 |
| No | 3443 (69.4) | 1929 (71) | 572 (54.5) | 942 (78.9) |  |
| Yes | 1518 (30.6) | 789 (29) | 477 (45.5) | 252 (21.1) |  |
| Malignant cancer, n (%) |  |  |  |  | < 0.001 |
| No | 4269 (86.1) | 2358 (86.8) | 843 (80.4) | 1068 (89.4) |  |
| Yes | 692 (13.9) | 360 (13.2) | 206 (19.6) | 126 (10.6) |  |
| Charlson comorbidity index | 7.1 ± 2.4 | 7.0 ± 2.4 | 7.9 ± 2.5 | 6.6 ± 2.2 | < 0.001 |
| APSIII | 63.4 ± 26.1 | 62.3 ± 26.4 | 69.2 ± 25.4 | 60.8 ± 25.5 | < 0.001 |
| SAPSII | 48.0 ± 13.2 | 47.7 ± 13.4 | 49.8 ± 12.8 | 46.8 ± 13.0 | < 0.001 |
| OASIS | 38.9 ± 9.3 | 38.6 ± 9.2 | 39.1 ± 9.5 | 39.1 ± 9.2 | 0.194 |
| SOFA | 7.4 ± 2.9 | 7.4 ± 2.9 | 7.6 ± 3.0 | 7.1 ± 2.9 | < 0.001 |
| Vasofree28, n (%) |  |  |  |  | < 0.001 |
| No | 1029 (20.7) | 490 (18) | 279 (26.6) | 260 (21.8) |  |
| Yes | 3932 (79.3) | 2228 (82) | 770 (73.4) | 934 (78.2) |  |
| Ventfree28, n (%) |  |  |  |  | < 0.001 |
| No | 1028 (20.7) | 489 (18) | 281 (26.8) | 258 (21.6) |  |
| Yes | 3933 (79.3) | 2229 (82) | 768 (73.2) | 936 (78.4) |  |

Abbreviations: bpm=beats per minute; SBP=Systolic Blood Pressure; DBP=Diastolic Blood Pressure; bpm=breaths per minute; WBC=white blood cell; BUN=Blood Urea Nitrogen; APS III=Acute Physiology Score III; SAPS II=Simplified Acute Physiology Score II; OASIS=Outcome Prediction in the Intensive Care Unit: Simplified Acute Physiology Score; SOFA=Sequential Organ Failure Assessment; min=the lowest value; max=the highest value
