# Supplementary material for: Association between hemoglobin dynamic trajectories and 28-day mortality in elderly patients with sepsis: A retrospective cohort study using the MIMIC-IV database
Source: PLoS One. 2026 May 4;21(5):e0327443. doi: 10.1371/journal.pone.0327443 (PMC13138669; doi:10.1371/journal.pone.0327443)
Supplement: S3 Table — (DOC) [file pone.0327443.s006.doc]

| **S3 Table. Univariate Cox Regression Analysis Results for 28-Day Mortality.** | | |
| --- | --- | --- |
| **Variable** | **HR (95%CI)** | **p** |
| Age | 1.03 (1.02,1.04) | < 0.001 |
| Hb1 | 0.95 (0.92,0.98) | 0.003 |
| Heart rate-mean | 1.01 (1.01,1.02) | < 0.001 |
| SBP-mean | 0.9945 (0.9896,0.9995) | 0.032 |
| DBP-mean | 1.0093 (1.0025,1.0162) | 0.008 |
| Resp rate-mean | 1.1 (1.09,1.12) | < 0.001 |
| Temperature-mean | 0.84 (0.75,0.94) | 0.002 |
| SpO2-mean | 0.9 (0.87,0.93) | < 0.001 |
| Glucose-mean | 1 (1,1) | < 0.001 |
| Platelets-min | 1.0013 (1.0007,1.0019) | < 0.001 |
| WBC-max | 1.0041 (1.0004,1.0078) | 0.028 |
| Anion gap-max | 1.05 (1.04,1.06) | < 0.001 |
| Bicarbonate-min | 0.97 (0.96,0.98) | < 0.001 |
| BUN-max | 1.01 (1.01,1.01) | < 0.001 |
| Calcium-min | 0.95 (0.89,1.02) | 0.174 |
| Chloride-min | 0.97 (0.96,0.98) | < 0.001 |
| Creatinine-max | 1.09 (1.06,1.12) | < 0.001 |
| Sodium-min | 1.0064 (0.9943,1.0187) | 0.3 |
| Potassium-max | 1.17 (1.09,1.25) | < 0.001 |
| INR-max | 1.11 (1.07,1.14) | < 0.001 |
| PT-max | 1.01 (1.01,1.02) | < 0.001 |
| APTT-max | 1.0046 (1.0029,1.0063) | < 0.001 |
| Lactate-max | 1.06 (1.03,1.08) | < 0.001 |
| Myocardial infarct | 1.05 (0.91,1.21) | 0.499 |
| Congestive heart failure | 1.31 (1.15,1.48) | < 0.001 |
| Peripheral vascular disease | 0.91 (0.77,1.08) | 0.284 |
| Cerebrovascular disease | 1.38 (1.18,1.61) | < 0.001 |
| Chronic pulmonary disease | 1.08 (0.95,1.24) | 0.253 |
| Liver disease | 1.9 (1.61,2.24) | < 0.001 |
| Diabetes | 1.03 (0.89,1.18) | 0.704 |
| Renal disease | 1.26 (1.11,1.44) | < 0.001 |
| Malignant cancer | 1.65 (1.41,1.93) | < 0.001 |
| Charlson comorbidity index | 1.15 (1.13,1.18) | < 0.001 |
| APSIII | 1.03 (1.03,1.03) | < 0.001 |
| SAPSII | 1.04 (1.03,1.04) | < 0.001 |
| OASIS | 1.07 (1.06,1.08) | < 0.001 |
| SOFA | 1.13 (1.1,1.15) | < 0.001 |
| Trajectoriy (Class 2 as ref) |  |  |
| 1 | 1.64 (1.42,1.91) | < 0.001 |
| 3 | 1.28 (1.1,1.5) | 0.001 |
