# Supplementary material for: Association between hemoglobin dynamic trajectories and 28-day mortality in elderly patients with sepsis: A retrospective cohort study using the MIMIC-IV database
Source: PLoS One. 2026 May 4;21(5):e0327443. doi: 10.1371/journal.pone.0327443 (PMC13138669; doi:10.1371/journal.pone.0327443)
Supplement: S4 Table — (DOC) [file pone.0327443.s007.doc]

| **S4 Table. The results of covariate selection.** | | | | | |
| --- | --- | --- | --- | --- | --- |
| **Term1** | **coeff1** | **Change.percentage1** | **VIF** | **collinearity** | **select** |
| Crude | 0.5 | Ref. | 3.169 | 0 | Ref. |
| Age | -0.53 | 5.9 | 1.011 | 0 | Yes |
| Hb1 | 0.42 | -15.5 | 3.126 | 0 | Yes |
| Resp rate-mean | 0.42 | -16.4 | 1.444 | 0 | Yes |
| Temperature-mean | 0.39 | -21.5 | 1.27 | 0 | Yes |
| Anion gap-max | 0.4 | -20.2 | 3.503 | 0 | Yes |
| Bicarbonate-min | 0.47 | -5.1 | 2.289 | 0 | Yes |
| BUN-max | 0.31 | -36.7 | 2.347 | 0 | Yes |
| Creatinine-max | 0.44 | -12.4 | 2.531 | 0 | Yes |
| Charlson comorbidity index | 0.37 | -25.3 | 2.469 | 0 | Yes |
| APSIII | 0.36 | -27.2 | 3.106 | 0 | Yes |
| OASIS | 0.47 | -4.9 | 2.424 | 0 | Yes |
| INR-max | 0.52 | 4 | 12.24 | 1 | Pending |
| PT-max | 0.51 | 3.4 | 12.516 | 1 | Pending |
| Heart rate-mean | 0.49 | -1.3 | 1.52 | 0 | No |
| SBP-mean | 0.5 | 1.4 | 1.316 | 0 | No |
| DBP-mean | 0.49 | -1.4 | 1.394 | 0 | No |
| SpO2-mean | 0.48 | -2.6 | 1.21 | 0 | No |
| Glucose-mean | 0.5 | 0.4 | 1.012 | 0 | No |
| Platelets-min | 0.47 | -4.7 | 1.301 | 0 | No |
| WBC-max | 0.5 | -0.3 | 1.116 | 0 | No |
| Chloride-min | 0.45 | -9.1 | 1.454 | 0 | No |
| Potassium-max | 0.48 | -3.7 | 1.202 | 0 | No |
| APTT-max | 0.52 | 3.8 | 1.109 | 0 | No |
| Lactate-max | 0.52 | 3.8 | 2.234 | 0 | No |
| Congestive heart failure | 0.48 | -3.4 | 1.298 | 0 | No |
| Cerebrovascular disease | 0.5 | 0.2 | 1.194 | 0 | No |
| Liver disease | 0.47 | -5.2 | 1.255 | 0 | No |
| Renal disease | 0.46 | -6.8 | 1.945 | 0 | No |
| Malignant cancer | 0.46 | -6.8 | 1.596 | 0 | No |
| SAPSII | 0.45 | -9.3 | 2.902 | 0 | No |
| SOFA | 0.49 | -1.8 | 2.661 | 0 | No |
